# Supplementary material for: Measuring arterial tortuosity in the cerebrovascular system using Time-of-Flight MRI
Source: medRxiv. 2024 Dec 26:2024.12.23.24319570. Preprint. [Version 1] doi: 10.1101/2024.12.23.24319570 (PMC11703313; doi:10.1101/2024.12.23.24319570)
Supplement: Supplement 1 [file NIHPP2024.12.23.24319570v1-supplement-1.pdf]

medRxiv preprint doi: https://doi.org/10.1101/2024.12.23.24319570; this version posted December 26, 2024. The copyright holder for this preprint (which was not certified by peer review) is the author/funder, who has granted medRxiv a license to display the preprint in perpetuity.
It is made available under a CC-BY 4.0 International license .

Supplementary Information

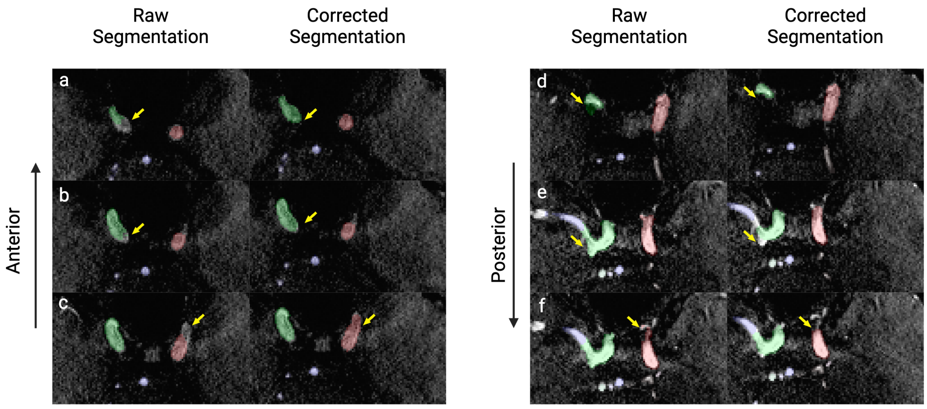

Figure S1. Quality Assurance example of eICAB segmentation

medRxiv preprint doi: https://doi.org/10.1101/2024.12.23.24319570; this version posted December 26, 2024. The copyright holder for this preprint (which was not certified by peer review) is the author/funder, who has granted medRxiv a license to display the preprint in perpetuity.
It is made available under a CC-BY 4.0 International license .

Supplementary Tables

Metric Name	Metric Symbol	Metric Formula
**Arc over Chord (AOC)**	τ	$\tau = \frac{L}{C}$
**Mean(Total) Curvature**	κ_m	$\kappa_m = \int_{t_1}^{t_2} \kappa(t)\, dt$
**Mean(Total) Squared Curvature**	κ_{ms}	$\kappa_{ms} = \int_{t_1}^{t_2} \kappa^2(t)\, dt$
**Normalized Root Mean Squared Curvature**	κ_{rms}	$\kappa_{rms} = \sqrt{\frac{1}{L} \int_{t_1}^{t_2} \kappa^2(t)\, dt}$

Table S1. Curvature and Tortuosity Metrics
